# The efficacy of licorice root extract on meat amino acid, fatty acid, vitamin, and mineral composition and productivity of quail

**DOI:** 10.14202/vetworld.2024.1017-1025

**Published:** 2024-05-09

**Authors:** Birzhan Nurgaliyev, Zhenis Kushmukhanov, Abzal Kenesovich Kereyev, Utegen Taubaev, Yerbol Sengaliyev, Svetlana Bayantassova, Ilana Abirova, Berik Satybaev, Aigerim Kozhayeva, Rinat Abdrakhmanov, Assel Paritova, Askhat Zhumabaev

**Affiliations:** 1 Department of Veterinary and Biological Safety, Institute of Veterinary Medicine and Animal Husbandry, West Kazakhstan Agrarian and Technical University named after Zhangir khan, Uralsk 090009, Republic of Kazakhstan; 2 Department of Veterinary Sanitation, Faculty of Veterinary and Animal Husbandry Technology, Saken Seifullin Kazakh Agro-Technical Research University, Astana 010011, Republic of Kazakhstan

**Keywords:** amino acids, licorice root extract, minerals, quails, vitamins

## Abstract

**Background and Aim:**

Poultry meat is an excellent animal protein source accessible to many low-income families in developing countries. It is also part of a balanced diet and contains valuable nutrients necessary for maintaining human health. The poultry sector implements improved processes to increase the quality and nutritional value of poultry meat. This study aimed to determine the influence of licorice root extract on the amino acid, fatty acid, vitamin, mineral composition, nutritional value, and productivity of quail meat.

**Materials and Methods:**

Two groups were formed from Japanese quails: A control group and one experimental group, each consisting of 50 individuals. Quails from both the experimental and control groups received the same complete diet. Quails in the experimental group had licorice root extract added to their water at a dosage of 10 g/L, starting from the age of 3 days to 42 days of growth. At 42 days of age, 30 birds from each group were slaughtered to examine their meat productivity and chemical composition. The quail carcasses were analyzed for the following parameters: Live weight, carcass weight, nutritional value, mineral substances, vitamin content, fatty acid composition, amino acid composition, and amino acid score.

**Results:**

This study demonstrated that quails in the experimental group receiving water with licorice extract exhibited higher indicators than those in the control group. Calcium (21.05%), magnesium (20.83%), and phosphorus (23.53%) were the most elevated mineral substances in the meat of the experimental birds. Vitamins E (22.22%) and C (20.0%) showed the greatest increase in vitamin content. The fatty acid composition parameters 17:0 margaric acid (8.16%), 18:3 linolenic acid (6.25%), and 20:4 arachidonic acid (4.49%) showed the highest increase. There was a clear increase in the amino acids valine (4.61%), lysine (4.32%), threonine (5.99%), tryptophan (4.87%), phenylalanine (5.87%), and cysteine (14.17%). The application of licorice root extract also positively impacted the amino acid score of quail meat, except for leucine, which remained within the range compared with the control group. Quails in the experimental group weighed 7.96% more live weight before slaughter than the controls. Moreover, the carcass weight was in favor of the experimental group (8.59%).

**Conclusion:**

The use of licorice root extract positively influences the quality and biological value of quail meat. Data on amino acids, fatty acids, vitamins, trace elements, and other important components of quail meat will significantly expand our understanding of the biological value of licorice root extract. These findings can be used in the formulation of balanced diets for children and adults and highlight the importance of this issue.

## Introduction

Poultry farming is one of the fastest-growing and most flexible sectors of animal husbandry. In particular, due to very high demand, it has expanded, consolidated, and gained a global character in countries with varying income levels. Rural poultry, in particular, plays an important role in the livelihood of a large number of farmers with limited resources, often representing their only asset [1–4]. The poultry sector in the Republic of Kazakhstan also has a leading position in the provision of high-quality meat products to the population. Poultry meat contains all the necessary nutrients that can be easily absorbed by the human body [5, 6].

The main criteria for poultry meat quality are biosecurity and nutritional value, which are ensured by biologically active additives of natural origin. Therefore, new feed additives are being introduced into veterinary practice to balance the diets of poultry, and their meat is becoming a beneficial dietary product for both children and adults [7–9].

Herbaceous plants and products derived from them are currently at the forefront of attention worldwide. The growing interest in these plants is due to their unique properties that can positively affect the quality of final poultry products, including carcass and meat characteristics [10–13].

A detailed analysis of the use of licorice root extract (*Glycyrrhiza glabra*) in poultry farming is provided in this publication. This plant belongs to legume family (*Fabaceae*). The use of licorice root extract in traditional medicine dates back over 4,000 years, and licorice root extract is promising in animal husbandry and poultry farming [14–16].

The use of medicinal plants and their derivatives, including licorice (*G. glabra*), has been extensively investigated. However, research on licorice exclusively is significantly limited [17].

In this study, the chemical composition and biological value of licorice root extract in poultry farming are analyzed. In addition, this study examines its impact on productivity indicators, carcass quality, and poultry meat production. This study contributes to the safe assessment and identification of new prospects for future studies investigating the beneficial properties of licorice root extract in poultry nutrition and its influence on meat quality.

This study aimed to determine the influence of licorice root extract on the amino acid, fatty acid, vitamin, mineral composition, nutritional value, and productivity of quail meat.

## Materials and Methods

### Ethical approval

The experiments were conducted in accordance with national and international laws based on the guidelines of the European Convention for the Protection of Vertebrate Animals used for Experimental and Other Scientific Purposes [18]. All procedures were discussed and approved during meetings of the Local Commission on Biological Ethics at the Zhangir Khan West Kazakhstan Agrarian and Technical University, Republic of Kazakhstan (Approval number: WKATU-3/2020, dated March 13, 2020).

### Study period and location

**Figure-1:**
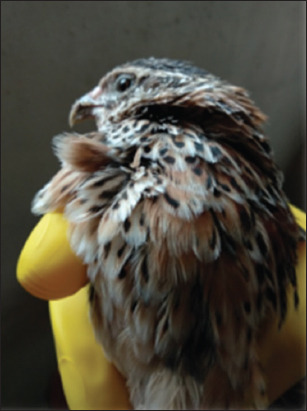
Japanese quails.

The study was conducted from April to June 2020 at the quail farm of the Higher School of Veterinary Biological Safety at the Zhangir Khan West Kazakhstan Agrarian and Technical University. This study focused on the Japanese quail breed (Figure-1). Quail meat quality was assessed in the laboratory of LLP “Nutritest” in the Republic of Kazakhstan.

### The quail and diet

The maintenance conditions for both groups were identical and met the requirements. During the observation period, the quails were clinically healthy; their behavior, physiological condition, and appearance in the study groups were identical and consistent with age-related changes.

### Experimental design

**Figure-2:**
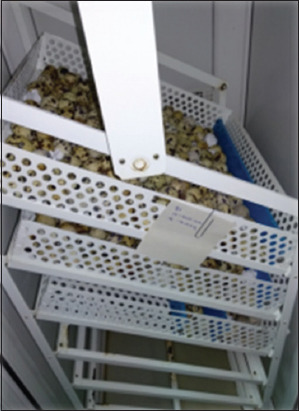
Incubation of quail eggs.

**Figure-3:**
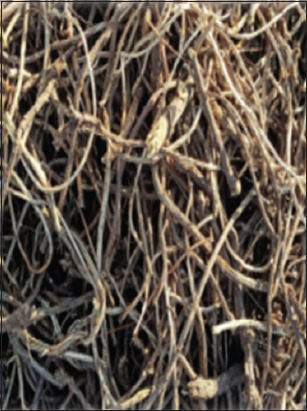
Licorice root.

**Figure-4:**
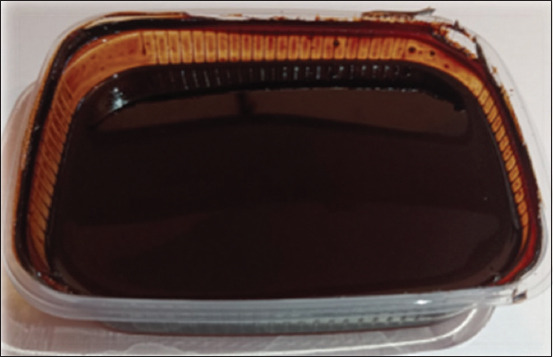
Licorice root extract.

Following the incubation of Japanese quail eggs (Figure-2), two groups were formed from newly hatched quails: A control group and one experimental group, each consisting of 50 individuals. The experimental and control groups received the same complete diet. In addition, the experimental group quails were supplemented with thick licorice root extract (Figures-3 and 4) (produced by LLP Licorice Priuralya, GOST 22840-77) [19] at a dosage of 10 g/L from the 3^rd^ day of age to the 42^nd^ day of cultivation. Three replications were performed for each group in this study.

At the age of 42 days, 30 birds from each group were slaughtered to study meat productivity and the chemical composition of the meat. Quail carcasses were subjected to the following parameters after slaughter: Live weight, carcass weight, nutritional value, mineral content, vitamin content, fatty acid composition, amino acid composition, and amino acid score.

### Determination of nutritional value

Moisture content in the meat was determined using the drying method according to GOST 9793-2016, “Meat and Meat Products: Method for Determination of Moisture” [20]. The Kjeldahl method was used to determine the protein content in the meat according to GOST 25011-81, “Meat and Meat Products: Methods for Determination of Protein” [21]. We determined the fat content according to GOST 23042-2015, “Meat and Meat Products: Methods for Determination of Fat” [22]: The ash content was determined according to GOST 31727-2012 (ISO 936:1998), “Meat and Meat Products: Method for Determination of the Mass Fraction of Total Ash” [23]. The energy value of poultry meat was calculated in accordance with GOST 34567-2019 [24].

### Determination of mineral content

Quantities of macroelements and microelements were determined in accordance with the following standards: Calcium, according to GOST R 55573-2013, “Meat and meat products. Determination of calcium by titrimetric methods” [25] and phosphorus, according to GOST 32009-2013 (ISO 13730:1996), “Meat and meat products. Spectrophotometric method (trilonometric method) for determining the mass fraction of total phosphorus” [26]. Sodium, potassium, and magnesium concentrations were determined by GOST R 55484-2013, “Meat and meat products. Determination of sodium, potassium, magnesium, and manganese by flame atomic absorption” [27]. Iron concentration was determined according to GOST 26928-86, “Food products.” Method for determining iron content [28].

### Determination of vitamin content

Vitamins were determined according to the “Guidelines for Methods of Quality Control and Safety of Biologically Active Food Supplements” (P 4.1.1672-2003, Chapter 2, Section 1) [29].

### Determination of fatty acid composition

The fatty acid composition of quail meat was determined according to the “Methodology of Gas Chromatographic Determination of Fatty Acids and Cholesterol in Food and Blood Serum MVI.MN. 1364-2000” [30].

### Determination of amino acid composition

The amino acid content of meat samples was analyzed using the “Method for the determination of amino acids in food using high-performance liquid chromatography MVI.MN.” 1363-2000 [31].

### Determination of amino acid score

Amino acid score was calculated using the following formula:

Amino acid score = (mg of amino acid in 1 g of test protein)/(mg of amino acid in reference pattern) 100 [32].

### Live weight and carcass weight

When the quails reached 42 days of age, 30 individuals were selected from each group and subjected to standard slaughter procedures in accordance with GOST R 52837-2007 “Slaughter poultry,” “Specifications” [33] and GOST R 54673-2011 for”Quail meat (carcass),” “Technical specifications” [34]. Subsequently, quail carcasses were anatomically dissected according to GOST R 52702-2006, “Chicken meat (carcasses of chickens, broiler-chickens, and their parts).” “Specifications” [35], we selected quail carcasses for chemical analysis.

### Statistical analysis

Numerical data were analyzed using Microsoft Excel 2016 (Microsoft Corp., Washington, U.S.). The results are presented as mean values ± standard errors of the mean. Student’s test was used to determine the significance of differences between mean values. Significant (p = 0.05), distinctly significant (p = 0.010), or very significant (p = 0.001) differences between the analyzed means are indicated by superscripts.

## Results

### Live weight and carcass weight

**Figure-5:**
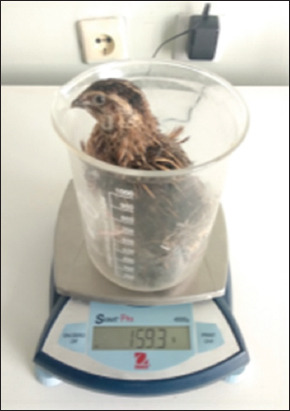
Weighing of quail carcasses.

**Figure-6:**
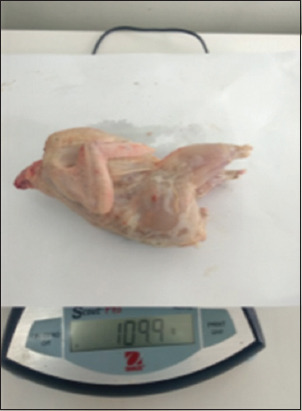
Weighing of quails.

The results of our studies showed that quails in the experimental group exhibited a higher live weight before slaughter, measuring 159.85 ± 2.68 g (p = 0.010), which was 7.96% greater than that of their control counterparts at 147.12 ± 2.15 g (p = 0.010). Similarly, in terms of carcass weight, the advantage was also on the side of the birds receiving the licorice root extract. The parameter under study reached 109.25 ± 2.96 (p = 0.010), surpassing the value of 99.86 ± 2.64 (p = 0.010) in the control group (Figures-5 and 6).

### Nutrient value of the quail meat

Table-1 presents the results of determining the nutritional value. The research results indicate that the nutritional value indicators of the quail meat to which licorice root extract was added were higher, except for moisture, compared with the control group. In the experimental group, protein, fat, and ash contents increased by 2.76%, 2.95%, and 22.22%, respectively. In addition, the energy value increased by 2.67%. Only moisture was reduced by 1.87% among the indicators.

**Table 1:** Nutritional value of quail meat (g/100 g).

Parameter and measurement	Control group	Experimental group	Effect of licorice root extract application
Proteins, g/100 g	18.1 ± 0.18*	18.6 ± 0.15*	Increased
Fats, g/100 g	16.9 ± 0.16*	17.4 ± 0.14*	Increased
Moisture, g/100 g	64.1 ± 0.71*	62.9 ± 0.67*	Decreased
Ash, g/100 g	0.9 ± 0.08***	1.1 ± 0.06***	Increased
Energy value, kcal/100 g	225 ± 3.42*	231 ± 3.58*	Increased

* =Significant at p ≤ 0.05,

*** =Very significant at p ≤ 0.001

### Mineral content in quail meat

We investigated sodium, potassium, calcium, magnesium, and phosphorus among the macronutrients. Among the micronutrients, we investigated iron. Analysis of the mineral composition of muscle tissue showed (Table-2) that when licorice root extract was applied, the content of sodium increased by 12.12%, potassium by 3.19%, calcium by 21.05%, magnesium by 20.83%, phosphorus by 23.53%, and iron by 3.23%.

**Table 2:** Mineral content in quail meat, per 100 g.

Parameter and measurement	Control group	Experimental group	Result of licorice root extract application
Sodium, mg	33 ± 0.97**	37 ± 1.10**	Increased
Potassium, mg	251 ± 4.13*	259 ± 4.07*	Increased
Calcium, mg	19 ± 0.31***	23 ± 0.32***	Increased
Magnesium, mg	24 ± 0.48***	29 ± 0.55***	Increased
Phosphorus, mg	17 ± 0.35***	21 ± 0.39***	Increased
Iron, mg	3.1 ± 0.06*	3.2 ± 0.05*	Increased

* =Significant at p ≤ 0.05,

** =Distinct significant at p ≤ 0.010,

*** =Very significant at p ≤ 0.001

### Vitamin content in quail meat

B1, B2, PP, and C were isolated from water-soluble vitamins. We investigated A and E from fat-soluble vitamins: The vitamin content in quail meat (Table-3) showed that the use of licorice root extract positively increased the vitamin content in the meat. The contents of vitamin A, vitamin E, vitamin B1, vitamin B2, vitamin PP, and vitamin C increased by 2.90%, 22.22%, 8.0%, 8.51%, 2.47%, and 20.0%, respectively.

**Table 3:** Content of vitamins in quail meat, per 100 g.

Parameter and measurement	Control group	Experimental group	Effect of licorice root extract application
A, mcg	6.9 ± 0.08*	7.1 ± 0.07*	Increased
E, mg	0.18 ± 0.31***	0.22 ± 0.0038***	Increased
B1, mg	0.25 ± 0.0035**	0.27 ± 0.0045**	Increased
B2, mg	0.47 ± 0.009**	0.51 ± 0.01**	Increased
PP, mg	8.1 ± 0.09*	8.3 ± 0.08*	Increased
C, mg	0.15 ± 0.003***	0.18 ± 0.002***	Increased

* =Significant at p ≤ 0.05,

** =Distinct significant at p ≤ 0.010,

*** =Very significant at p ≤ 0.001

### Fatty acid composition of quail meat

Up to ten major fatty acids were identified in the analyzed samples. Our research findings (Table-4) indicate that the application of licorice root extract leads to an increase in saturated fatty acids (SFA) in poultry meat: 14:0 myristic acid increased by 3.21%, 16:0 palmitic acid by 0.29%, 17:0 margaric acid by 8.16%, and 18:0 stearic acid by 1.33%. We also observed an increase in monounsaturated fatty acids (16:1 palmitoleic acid increased by 2.92%, 18:1 oleic acid increased by 0.12%, and 20:1 gadoleic acid increased by 4.24%) in the experimental group. Licorice extract also increased polyunsaturated fatty acids (PUFAs): 18:2 linoleic acid increased by 0.84%, 18:3 linolenic acid increased by 6.25%, and 20:4 arachidonic acid increased by 4.49%.

**Table 4:** Fatty acid composition of quail meat, mg/100 g.

Parameter and measurement	Control group	Experimental group	Effect of licorice root extract application
Saturated fatty acid, including	4506 ± 28.07*	4541 ± 30.13*	Increased
C_14:0_ Myristic acid, mg/100 g	187 ± 3.65*	193 ± 3.45*	Increased
C_16:0_ Palmitic acid, mg/100 g	3069 ± 36.34	3078 ± 32.28	Increased
C_17:0_ Margaric acid, mg/100 g	49 ± 0.95**	53 ± 0.94**	Increased
C_18:0_ Stearic acid, mg/100 g	1201 ± 11.82*	1217 ± 12.07*	Increased
Monounsaturated fatty acid, including	5932 ± 40.14*	5948 ± 42.63*	Increased
C_16:1_ Palmitic acid, mg/100 g	137 ± 2.65*	141 ± 2.78*	Increased
C_18:1_ Oleic acid, mg/100 g	5677 ± 56.89	5684 ± 56.63	Increased
C_20:1_ Gadoleic acid, mg/100 g	118 ± 2.34*	123 ± 2.43*	Increased
Polyunsaturated fatty acid, including	3070 ± 23.52*	3106 ± 25.54*	Increased
C_18:2_ Linoleic acid, mg/100 g	2853 ± 23.58	2877 ± 22.64	Increased
C_18:3_ Linolenic acid, mg/100 g	128 ± 2.51**	136 ± 2.52**	Increased
C_20:4_ Arachidonic acid, mg/100 g	89 ± 1.69**	93 ± 1.77**	Increased
Total fatty acids	13508 ± 75.67*	13595 ± 78.11*	Increased

* =Significant at p ≤ 0.05,

** =Distinct significant at p ≤ 0.010

### Amino acid composition of quail meat

Table-5 presents the amino acid composition of the quail meat. All amino acids were higher in the experimental group than in the control group. The application of licorice root extract increased the levels of indispensable amino acids: Valine increased by 4.61%, isoleucine by 3.01%, leucine by 2.49%, lysine by 4.32%, methionine by 0.97%, threonine by 5.99%, tryptophan by 4.87%, and phenylalanine by 5.87%. Replaceable amino acids were also elevated in the experimental group: alanine by 0.46%, arginine by 1.12%, aspartic acid by 0.55%, histidine by 2.44%, glycine by 0.59%, glutamic acid by 3.48%, hydroxyproline by 3.14%, proline by 1.02%, serine by 1.54%, tyrosine by 0.62%, and cysteine by 14.17%.

**Table 5:** Amino acid composition of quail meat, mg/100 g.

Parameter and measurement	Control group	Experimental group	Effect of licorice root extract application
Essential amino acids, including	7368 ± 62.16*	7659 ± 65.52*	Increased
Valine, mg/100 g	954 ± 12.48*	998 ± 13.15*	Increased
Isoleucine, mg/100 g	865 ± 10.57*	891 ± 9.58*	Increased
Leucine, mg/100 g	1608 ± 17.12*	1648 ± 15.29*	Increased
Lysine, mg/100 g	1481 ± 22.87*	1545 ± 21.93*	Increased
Methionine, mg/100 g	516 ± 4.24	521 ± 4.13	Increased
Threonine, mg/100 g	801 ± 17.01**	849 ± 14.98**	Increased
Tryptophan, mg/100 g	308 ± 5.18*	323 ± 5.25*	Increased
Phenylalanine, mg/100 g	835 ± 12.76**	884 ± 13.93**	Increased
Non-essential amino acids, including	10690 ± 91.45*	10877 ± 98.97*	Increased
Alanine, mg/100 g	1098 ± 15.29	1103 ± 15.18	Increased
Arginine, mg/100 g	1072 ± 8.12*	1084 ± 9.17*	Increased
Asparagine, mg/100 g	1649 ± 15.41	1658 ± 12.38	Increased
Histidine, mg/100 g	328 ± 3.05*	336 ± 2.84*	Increased
Glycine, mg/100 g	1184 ± 12.19	1191 ± 11.23	Increased
Glutamic, mg/100 g	2903 ± 32.54*	3004 ± 28.57*	Increased
Hydroxyproline, mg/100 g	191 ± 2.91*	197 ± 2.60*	Increased
Proline, mg/100 g	782 ± 7.19*	790 ± 6.94*	Increased
Serine, mg/100 g	715 ± 5.91*	726 ± 6.41*	Increased
Tyrosine, mg/100 g	647 ± 7.13	651 ± 6.81	Increased
Cysteine, mg/100 g	120 ± 1.95***	137 ± 1.41***	Increased
Total amino acids	18058 ± 166.18*	18536 ± 171.47*	Increased

* =Significant at p ≤ 0.05,

** =Distinct significant at p ≤ 0.010,

*** =Very significant at p ≤ 0.001

### Amino acid score of quail meat

Table-6 presents the results of determining the amino acid scores for quail meat. The findings of this study demonstrate that the application of licorice root extract also has a positive impact on the amino acid score of quail meat. In the experimental group, isoleucine, lysine, methionine + cysteine, phenylalanine + tyrosine, threonine, tryptophan, and valine increased by 0.84%, 1.34%, 1.0%, 1.47%, 2.7%, 2.35%, and 1.9%, respectively. The leucine levels remained within the range compared with the control group values.

**Table 6:** Amino acid score of quail meat, %.

Parameter and measurement	Control group	Experimental group	Effect of licorice root extract application
Isoleucine, %	119 ± 1.21	120 ± 1.19	Increased
Leucine, %	127 ± 2.02	127 ± 1.75	Unchanged
Lysine, %	149 ± 1.16*	151 ± 1.35*	Increased
Methionine+Cysteine, %	100 ± 1.39*	101 ± 1.28*	Increased
Phenylalanine+Tyrosine, %	136 ± 2.01*	138 ± 2.03*	Increased
Threonine, %	111 ± 1.91*	114 ± 1.85*	Increased
Tryptophan, %	170 ± 2.40*	174 ± 2.27*	Increased
Valine, %	105 ± 2.14*	107 ± 2.23*	Increased

* =Significant at p ≤ 0.05

## Discussion

Herbaceous plants and products derived from them have become widely popular, and their importance is recognized around the world [36]. Increased interest in these plants is due to their characteristics, which can have a positive impact on the performance of end products in poultry production [37, 38]. The use of plant extracts in poultry production is expanding due to their positive effects on digestion, appetite stimulation, and improvement of various physiological functions. These effects contribute to alleviating health problems and improving overall performance [39]. In regions where this plant grows widely and is readily available, its use in poultry diets can be economically beneficial [40].

### Live weight and carcass weight

The addition of licorice root extract to water has a positive effect on the productivity of the poultry. The experimental group of quails exhibited a greater live mass before slaughter compared with their control counterparts. In addition, licorice root extract had an advantage in carcass mass of birds. Hosny *et al*. [41] and Frankič *et al*. [42] reported that the inclusion of licorice root extract in poultry water had a positive impact on the live weight gain of birds and carcass weight. In addition, licorice contains components that stimulate appetite and improve digestion processes. Salary *et al*. [43] and Hosseini *et al*. [44] observed the greatest body weight gain when licorice was added to drinking water, whereas the supplementation of licorice in the diet yielded superior results in birds where licorice was used. Incorporating licorice into poultry diets is a safe and effective method that enhances poultry productivity. In their study, Ocampo *et al*. [45] emphasized that birds receiving glycyrrhizic acid demonstrated better body weight gain, final weight, and lower mortality compared with birds not receiving this substance. Therefore, they concluded that the use of glycyrrhizic acid in drinking water could improve the health and productivity of poultry. In the present study, the survival rate of quails in the experimental group was 100%, whereas that in the control group was 10%.

### The nutritional value of quail meat

To properly interpret questions relating to the nutritional value of meat products, it is necessary, in particular, to have an objective understanding of the overall chemical composition of the meat raw material. The general chemical composition of meat raw material consists of mass fractions of moisture, fat, protein, and ash in most scientific and technical information sources [46, 47]. Our study showed that the nutritional value of the meat of quails given licorice root extract was higher, except for moisture than that in the control group. Proteins, fats, and energy values increased in the experimental group, and the greatest increase was observed in ash. Moisture values of the experimental group decreased.

### Mineral content in the meat of quail

Mineral substances play a plastic role in the vital processes of the body and participate in the metabolism of almost any tissue. However, they play a particularly important role in the construction of bone tissue, where elements such as phosphorus and calcium are predominant. The research results indicate that the use of licorice root extract contributes to an increase in the mineral content of quail meat, particularly calcium, magnesium, phosphorus, and sodium. Badr *et al*. [48] and Mohammed *et al*. [49] studied the mineral composition of licorice and its extract and found that it is rich in mineral substances. Calcium, sodium, phosphorus, potassium, and iron were the most common minerals found in licorice and its extract. When licorice root extract is used in feeding, it saturates the bird’s body with mineral substances. In general, quail meat is an important source of many essential minerals when licorice root extract is included in its diet and can be incorporated into a variety of diets to provide the body with the necessary trace elements.

### Vitamin content in quail meat

The deficiency and absence of vitamins in meat lead to hypovitaminosis and avitaminosis, specific pathological manifestations. In this study, the most significant increase was observed in vitamin E and vitamin C. According to Babich *et al*. [50], licorice extract contains a large amount of vitamins and is very rich in vitamin C, B2, and B6. The phytochemical analysis in the studies by Pastorino *et al*. [51] revealed that licorice is also rich in various vitamins, and its pharmacological action plays a crucial role in maintaining the health of the organism. The addition of licorice root extract to poultry water provides the birds with essential vitamins that contribute to their health and overall well-being. Incorporation of poultry meat enriched with vitamins into the diet can be a beneficial way of providing the human body with the necessary vitamins, in particular due to its excellent absorbability and taste.

### Fatty acid composition of quail meat

The main components of lipids are fatty acids. SFAs are used by the body as energy sources. Animal fats generally contain more SFAs than plant fats [52]. The greatest increases after the application of licorice root extract were observed in the presence of 17:0 margaric acid, 20:1 gadoleic acid, 18:3 linolenic acid, and 20:4 arachidonic acid extract. Zamaria [53] demonstrated that fatty acids constitute a significant portion of the components in the G. extract *Glabra*, providing cells with energy and serving as substrates for lipid synthesis. Studies by El-Saber Batiha *et al*. [54] indicated that the bioactive chemical components of licorice extract also enhance fatty acids, exerting a beneficial effect on biochemical processes in the body. Ahmed *et al*. [55] suggested that herbs in their natural or fermented form may alter the composition of fatty acids in muscle and increase the concentration of PUFAs. PUFAs, such as linoleic, linolenic, and arachidonic acids, are of particular importance because they are constituents of cell membranes and other structural elements of tissues and perform several crucial functions in the body, such as supporting normal growth and metabolism and vascular elasticity. PUFAs cannot be synthesized in the human body and are therefore essential, similar to certain amino acids and vitamins.

### Amino acid composition of quail meat

Proteins are one of the most valuable components of food and are involved in important bodily functions. The main importance of proteins lies in their indispensability to other nutrients. Dietary proteins are broken down into amino acids in the human body [56, 57]. The results showed that the addition of licorice extract to the water of birds led to a greater increase in indispensable amino acids compared with replaceable amino acids. Amino acids with the highest increase were valine, lysine, threonine, tryptophan, phenylalanine, and cysteine. Insufficient, indispensable amino acids may prevent the growth and development of the organism. Histidine and cysteine are also considered indispensable in infants. Nedil’ko and Yanitskaya [58] reported the biological value of licorice grass and a broad spectrum of pharmacological activity associated with its relatively high amino acid content. These findings are consistent with those of Bazekin *et al*. [59] and Fu *et al*. [60], who demonstrated that licorice root extract has a favorable influence on the chemical and amino acid composition of the meat. As poultry meat supplemented with licorice root extract contains a wide range of amino acids, it is a crucial source of protein and nutrients for humans. Incorporation of this meat into the diet contributes to providing the body with the essential amino acids necessary for healthy functioning.

### Amino acid score of quail meat

The quality of protein depends on the presence of a complete set of essential amino acids in a specific ratio, both for themselves and for non-essential amino acids. 1 g of protein, when oxidized in the body, yields 4 kcal. Animal products, such as meat, are a source of complete protein and contain a full set of essential amino acids in sufficient quantities for protein biosynthesis in the human body. Animal proteins are absorbed by the body at a rate of 93%–96% [61–66]. To express the biological value of protein products, a method such as the amino acid (chemical) score method is used based on comparing the results of determining the amino acid composition of the investigated product with the “ideal” protein. In 2007, the Joint Expert Committee of the Food and Agriculture Organization of the United Nations (FAO) and the World Health Organization (WHO) proposed the amino acid composition of the “ideal” protein for calculating the amino acid score (Joint WHO/FAO/UNU Expert Consultation, 2007) [67–69]. Poultry meat treated with licorice root extract has a high amino acid content, indicating excellent quality. It is characterized by a complete and high-quality protein with good digestibility and the ability to provide all essential amino acids [70].

## Conclusion

It is well known that the quality of poultry meat depends largely on the nutrition and maintenance conditions. The addition of licorice root extract to birds’ drinking water stimulates the growth of quails and enhances the meat value. The application of licorice root extract in the experimental group’s water led to an increase in the live and carcass weights of the birds compared with the control group’s weights. In quail meat containing licorice root extract, there was an increase in all the essential substances necessary for human nutrition. Therefore, meat is a significant source of indispensable amino acids, fatty acids, vitamins, and minerals in optimal quantitative and qualitative proportions. The enrichment of poultry meat with substances beneficial to human health is an interesting question for future poultry meat production. In view of the shift in consumer behavior, the importance of this issue is even more pronounced. The results of this study underscore the beneficial properties and potential applications of licorice root extracts. Licorice root extract has a positive effect on the growth, productivity, and quality of poultry meat. These conclusions will be of great interest to scientists, veterinarians, and experts in the poultry industry.

## Authors’ Contributions

BN, ZK, UT, and AKK: Designed the study and analyzed the data. ZK, YS, BS, AK, AZ, and RA: Conducted the study. SB, IA, and AP: Recorded and analyzed the data. BN, AKK, and AP: Coordinated the study, wrote, and revised the manuscript. All authors have read, reviewed, and approved the final manuscript.
